# Meta-analysis of early-life antibiotic use and allergic rhinitis

**DOI:** 10.1515/med-2022-0459

**Published:** 2022-11-04

**Authors:** Xiang Liu, Rongrong Wu, Yong Fu, Wenxin Chen, Yang Chen, Yangyan Yan, Jing Bi, Jia Liu

**Affiliations:** Department of ENT and Head & Neck Surgery, Hangzhou First Hospital Affiliated to Zhejiang University School of Medicine, Hangzhou, 310003, Zhejiang, China; Department of ENT and Head & Neck Surgery, The Children’s Hospital Zhejiang University School of Medicine, 3333 Bingsheng Road, Hangzhou, 310051, Zhejiang, China

**Keywords:** antibiotics, allergic rhinitis, early life, meta-analysis

## Abstract

This meta-analysis aimed to investigate the correlation between early-life antibiotic use and allergic rhinitis. PubMed, Embase, and the Cochrane Central Register of Controlled Trials databases were searched for available studies. Eighteen studies covering 1,768,874 children were included. Early-life antibiotics were associated with an increased incidence of allergic rhinitis (effect size (ES) = 5.00, 95% confidence interval [CI]: 4.88–5.13; *I* ^2^ = 95.7%, *P*_heterogeneity_ <0.001). In Asia, Europe, and the USA, the incidence of allergic rhinitis in the antibiotic group was higher than that in the no medication group (Asia: ES = 3.68, 95% CI: 3.38–4.01; Europe: ES = 3.20, 95% CI: 3.00–3.42; USA: ES = 3.68, 95% CI: 2.74–4.95). Compared with the no medication group, children who received antibiotics in the first 1 week of life (ES = 5.75, 95% CI: 2.18–15.18), first 1 year of life (ES = 3.37, 95% CI: 3.20–3.55; *I* ^2^ = 64.2%, *P*_heterogeneity_ = 0.001), or first 3 years of life (ES = 5.21, 95% CI: 2.42–11.19) had a higher incidence of allergic rhinitis. No individual study influenced the estimates of the meta-analysis. The funnel plot showed moderate symmetry and low publication bias. In conclusion, the use of antibiotics in early life was associated with allergic rhinitis. Still, most included studies analyzed antibiotic exposure as a dichotomous variable, without information on the type and dosage of antibiotics.

## 1 Introduction

Antibiotics can prevent and cure many infectious diseases, but their irrational use will lead to the emergence of drug-resistant bacteria, aggravation of infection, damage to the whole body, and severe harm to human health [1,2,3]. Children’s central nervous and cardiovascular systems are immature and sensitive to drugs. Therefore, the unreasonable use of antibiotics will cause more severe damage to children than to adults [4,5]. The use of antibiotics will change the intestinal flora, imbalance the intestinal flora in early life, cause a lack of stimulation of normal beneficial intestinal flora and affect the development of immune function in early life, which might be closely related to the occurrence and development of allergic diseases [6,7].

Allergic diseases, such as asthma, atopic dermatitis, and allergic rhinitis, are common worldwide, imposing a huge economic burden on society with respect to healthcare costs [2]. Therefore, preventing the occurrence and development of allergic diseases in children is essential. Allergic rhinitis is an allergic disease occurring in nasal mucosa with main clinical manifestations as sneezing, itching, and stuffy nose [8,9]. Some patients have itching eyes and pharynx [8,9]. Presently, about 600 million individuals worldwide suffer from allergic rhinitis, and the incidence is increasing annually [8,9,10].

Several studies analyzed the correlation between antibiotics and allergic rhinitis in early life. An early study by Celedon et al. [11] demonstrated a correlation between antibiotics and allergic rhinitis in early life. On the other hand, Fsadni et al. [6] reported a limited association between antibiotics and allergic rhinitis in early life. Therefore, a meta-analysis was performed to evaluate the association between antibiotics and allergic rhinitis in early life.

## 2 Methods

### 2.1 Databases and search strategies

PubMed, Embase, and the Cochrane Central Register of Controlled Trials databases were searched for available papers published up to June 2021. Two independent investigators carried out the initial search, deleted the duplicate records, screened the titles and abstracts for relevance, and identified the publications to be excluded or required further assessment. Next, the investigators reviewed the full-text articles for inclusion. In addition, the references of the retrieved articles and previous reviews were manually checked to identify additional eligible studies. The keywords were anti-bacterial agents AND (rhinitis, allergic, OR rhinitis, allergic, seasonal) AND cohort (Table A1). These keywords were used in all possible combinations to retrieve the maximum number of articles. Any discrepancy or disagreement in the study selection process was solved through discussion until a consensus was reached.

This study is a meta-analysis; the data in the article are from published articles, so ethical approval was waived, and informed consent was not applicable.

### 2.2 Inclusion and exclusion criteria

Studies were included if:

(a) They were considered cohort studies or cross-sectional studies.
(b) They compared antibiotics vs no medications.
(c) They involved patients with allergic rhinitis.

Studies were excluded if:

(a) They were case reports, meta-analyses, or letters to the editors.
(b) No comparisons were made between antibiotics and no medications.
(c) Patients had other diseases.
(d) There were duplicates.

### 2.3 Data extraction and review

After selection, two authors analyzed the studies, which data were extracted for the following information: article identification (author, year, study location, and study design), sample characteristics (number of patients in each study and age), the diagnosis of AR, timing of antibiotic exposure, and influencing factors. Any disagreement was discussed, and a third reviewer was consulted when necessary.

The methodological quality of the cohort and cross-sectional studies was evaluated using the Newcastle–Ottawa Scale (NOS) [12]. The quality score of the studies was calculated, with a maximum score of nine points for cohort studies and seven points for cross-sectional studies. This study adheres to the Equator guidelines [13].

### 2.4 Statistical analysis

STATA SE 14.0 (StataCorp, College Station, TX, USA) was used to calculate the odds ratios (ORs) with 95% confidence intervals (CIs). The significance and the extent of statistical heterogeneity were calculated using the *Q*-test and *I* ^2^ index, respectively. The random-effects model was applied if the *P*-value for the test of heterogeneity was <0.10. ORs were calculated for each analysis with the corresponding 95% CIs. Funnel plots were used to detect the possibility of publication bias by evaluating the asymmetry [14]. In addition, a sensitivity analysis was performed to identify individual study effects on pooled results and test the reliability of the results [14].

## 3 Results

### 3.1 Search results

**Figure 1:**
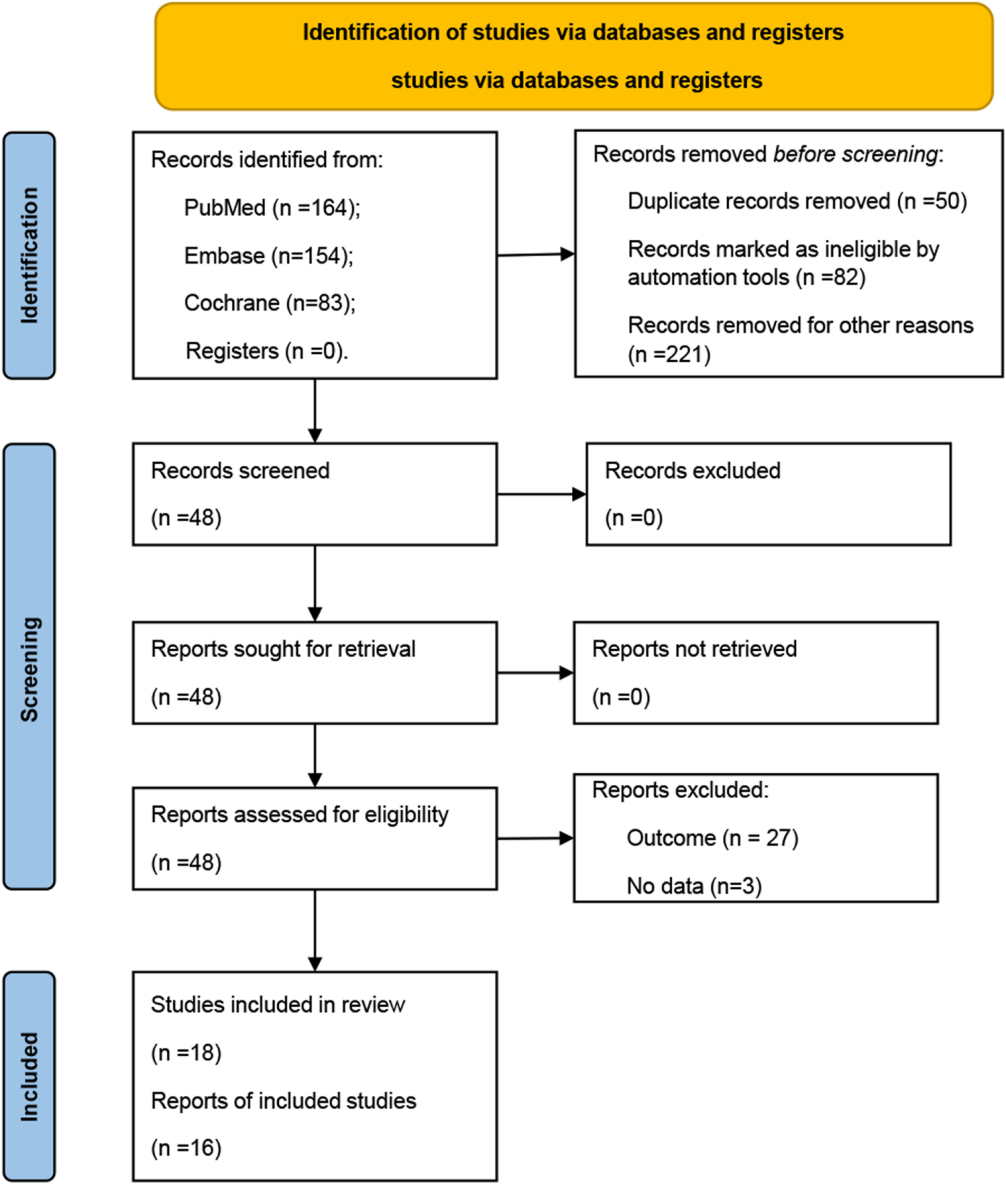
Schematic representation of study identification, inclusion, and exclusion.

The electronic search retrieved 401 articles; 353 reports were removed before screening (Figure 1). Forty-eight reports were screened, retrieved, and assessed for eligibility. Thirty were excluded because of the outcome (*n* = 27) and no data available (*n* = 3). Finally, 18 studies fulfilled the inclusion criteria.

### 3.2 Main features of the studies

Table 1 summarizes the type of study reported and the total number of patients associated with each group. There were nine cohort studies [1,15,16,17,18,19,20,21,22] and nine cross-sectional studies [23,24,25,26,27,28,29,30,31] for a total of 1,768,874 children.

**Table 1:** Characteristics of the included studies

Author, year	Design	Country	Source of subjects	The diagnosis of AR	Sample size	Age (years)	Male (%)	Timing antibiotic exposure	Influencing factor
Ortqvist et al., 2014 [1]	Prospective cohort	Sweden	Population	Questionnaire	4,033	8	61.10	In the first week of life	Maternal age, paternal education, rural residence first year, living on a farm with animals at preschool age, daily outdoor time, family history of allergic disease, exposure to cat and dog first year, frequent consumption of fish and fermented food, allergic disease first year, maternal medication during pregnancy, neonatal antibiotics, large for gestational age, pacifier, and sex
Aversa et al., 2021 [15]	Retrospective cohort	USA	Population	ICD-9/10	14,572	/	51.78	In the first 2 years of life	Male sex, birth weight, ethnicity, and cesarean section, age, education, smoking, and antibiotic use during pregnancy
Celedón et al., 2002 [16]	Prospective cohort	USA	Population	Questionnaire	498	/	53.80	In the first 1 year of life	Sex, household income, and history of hay fever (ever) in either parent
Harris et al., 2007 [17]	Prospective cohort	UK	Population	Standard questions (ISAAC)	641	8	53.35	In the first 5 years of life	Maternal atopy, paternal atopy, birth order, and current exposure to cigarette smoke
Ho and Wu, 2021 [23]	Cross-sectional	China	Population	Questionnaire (modified ISAAC)	23,630	6–8	51.80	In the first 1 year of life	Sex, bronchiolitis before the age of two, older siblings, diagnosed asthma, diagnosed eczema
Kim et al., 2018 [24]	Cross-sectional	Korea	Population	ICD-10	1,541,869	<10, 81.28%	51.30	/	Basic, age and sex, inpatient and outpatient days, income, and place of residence
Mai et al., 2010 [18]	Prospective cohort	Sweden	Population	BAMSE questionnaire	4,089	4–8	/	In the first 1 year of life	Sex, young maternal age, older sibling, maternal smoking, parental allergy, and exclusive breast-feeding
Metzler et al., 2019 [19]	Prospective cohort	European	Population	Questionnaire	1,019	6	51.03	In the first 1 year of life	Farmer, center, parental atopic status, gender, smoking during pregnancy, number of siblings, pets (dogs and cats) during pregnancy, cesarean section, maternal education
Mitre et al., 2018 [20]	Retrospective cohort	USA	Population	ICD-9-CM	131,708	/	53.90	In the first 0.5 years of life	Prematurity, cesarean delivery, sex, the other drug classes, and any significant first-order interaction terms
Ni et al., 2019 [21]	Retrospective cohort	USA	Population	ICD-9/10	2,398	5.7	51.00	In the first 1 year of life, lifetime	Race/ethnicity, age, sex, delivery method, prematurity, birth weight, NICU admission status, and socioeconomic status
Penaranda et al. [25]	Cross-sectional	Colombia	Population	Self-reported	7,085	6–7, 3,256/13–14,3829	45.70	In the first 1 year of life	Asthma symptoms in the last 12 months, atopic dermatitis symptoms in the last 12 months, use of acetaminophen in the first year of life, use of acetaminophen in the last 12 months
Sultesz et al., 2020 [26]	Cross-sectional	Hungary	Population	Core questions of ISAAC Phase I	3,836	6–12	48.40	In the first 1 year of life	/
Sultész et al., 2010 [27]	Cross-sectional	Hungary	Population	Core questions of ISAAC Phase I	3,933	6–12	50.20	In the first 1 year of life	/
Tamay et al., 2007 [28]	Cross-sectional	Turkey	Population	ISAAC questionnaire	2,387	6–12	49.64	In the first 1 year of life	Family history of atopy, the presence of physician-diagnosed eczema or food allergy, frequent upper airway infections and sinusitis, history of adenoidectomy, antibiotic or paracetamol use in the first year of life, cat or dog ownership in the first year of life, home dampness, exposure to diesel trucks, perianal redness
Tong et al., 2020 [29]	Cross-sectional	China	Population	ISAAC, ECRHS, SFAR	5,550	6–12	53.90	In the past 5 years	Gender, age, family history of allergy, air purifier
Yamamoto-Hanada et al., 2017 [22]	Prospective cohort	Japan	Hospital	Questionnaire	902	5	51.00	Younger than 3 years	Maternal history of allergy, maternal age at pregnancy, maternal smoking during pregnancy, mode of delivery, gestational age at delivery, daycare attendance, number of previous live births, bronchitis, and sex of the child
Yang et al., 2014 [30]	Cross-sectional	Korea	Population	ISAAC questionnaire	7,389	13.9	44.10	Antibiotic use in infancy	Age, sex, BMI, parental history of allergic rhinitis, each school, and household income
Zou et al., 2020 [31]	Cross-sectional	China	Population	ISAAC	13,335	4–6	50.60	In the first 1 year of life	Child’s age, sex, district, family history of atopy, breastfeeding, early home decoration, first-year pet-keeping, first-year environmental tobacco smoke (ETS), and first-year home dampness-related exposures

AR, allergic rhinitis; ISAAC, International Study of Asthma and Allergies in Childhood; ECRHS, European Community Respiratory Diseases Survey; BAMSE, Barn (Children) Allergy Milieu Stock-holm Epidemiology; SFAR, the Score for Allergic Rhinitis.

### 3.3 Quality assessment

**Figure 2:**
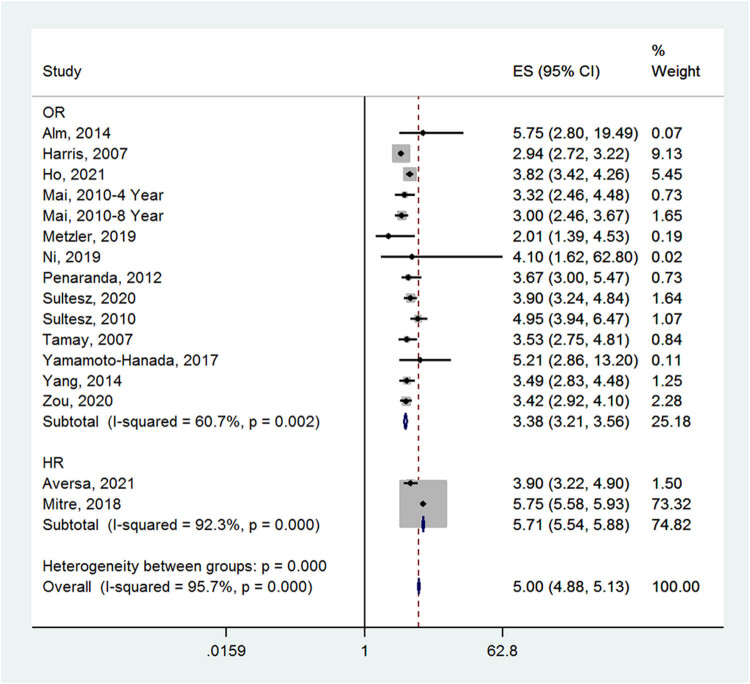
Forest plot for the incidence of allergic rhinitis in the antibiotic and no medication groups.

The quality assessment of the included studies is presented in Figure 2. Among the cohort studies, three scored seven points, one scored eight points, and five scored nine points. Among the cross-sectional studies, three scored six points, and six scored seven points (Table 2).

**Table 2:** Quality evaluation using the NOS scale

	Representativeness of the exposed cohort	Selection of the non-exposed cohort	Ascertainment of exposure	Demonstration that outcome of interest was not present at the start of the study	Comparability of cohorts based on the design or analysis	Assessment of outcome	Was follow-up long enough for outcomes to occur	Adequacy of follow-up of cohorts	Total quality scores
**Cohort studies**									
Ortqvist et al. [1]	*	*	*	*	**	*	*	*	9
Aversa et al. [15]	*	*	*	*	**	*	/	/	7
Celedón et al. [16]	*	*	*	*	**	*	*	*	9
Harris et al. [17]	*	*	*	*	**	*	*	*	9
Mai et al. [18]	*	*	*	*	**	*	*	*	9
Metzler et al. [19]	*	*	*	*	**	*	*	*	9
Mitre et al. [20]	*	*	*	*	**	*	/	/	7
Ni et al. [21]	*	*	*	*	**	*	/	/	7
Yamamoto-Hanada et al. [22]	/	*	*	*	**	*	*	*	8
**Cross-sectional studies**									
Ho and Wu [23]	*	*	*	**	*	*	7		
Kim et al. [24]	*	*	*	**	*	*	7		
Penaranda et al. [25]	*	*	/	**	*	*	6		
Sultesz et al. [26]	*	*	*	*	*	*	6		
Sultesz et al. [27]	*	*	*	*	*	*	6		
Tamay et al. [28]	*	*	*	**	*	*	7		
Tong et al. [29]	*	*	*	**	*	*	7		
Yang et al. [30]	*	*	*	**	*	*	7		
Zou et al. [31]	*	*	*	**	*	*	7		

### 3.4 Results of the meta-analysis

#### 3.4.1 Meta-analysis of the incidence of allergic rhinitis

Fifteen studies entered the meta-analysis of the incidence of allergic rhinitis. Early-life antibiotics were associated with an increased incidence of allergic rhinitis in children (effect size (ES) = 5.00, 95% CI: 4.88–5.13; *I* ^2^ = 95.7%, *P*_heterogeneity_ < 0.001) (Figure 2). The incidence of allergic rhinitis in the antibiotic group was higher than that of the no medication group.

#### 3.4.2 Subgroup meta-analysis

**Figure 3:**
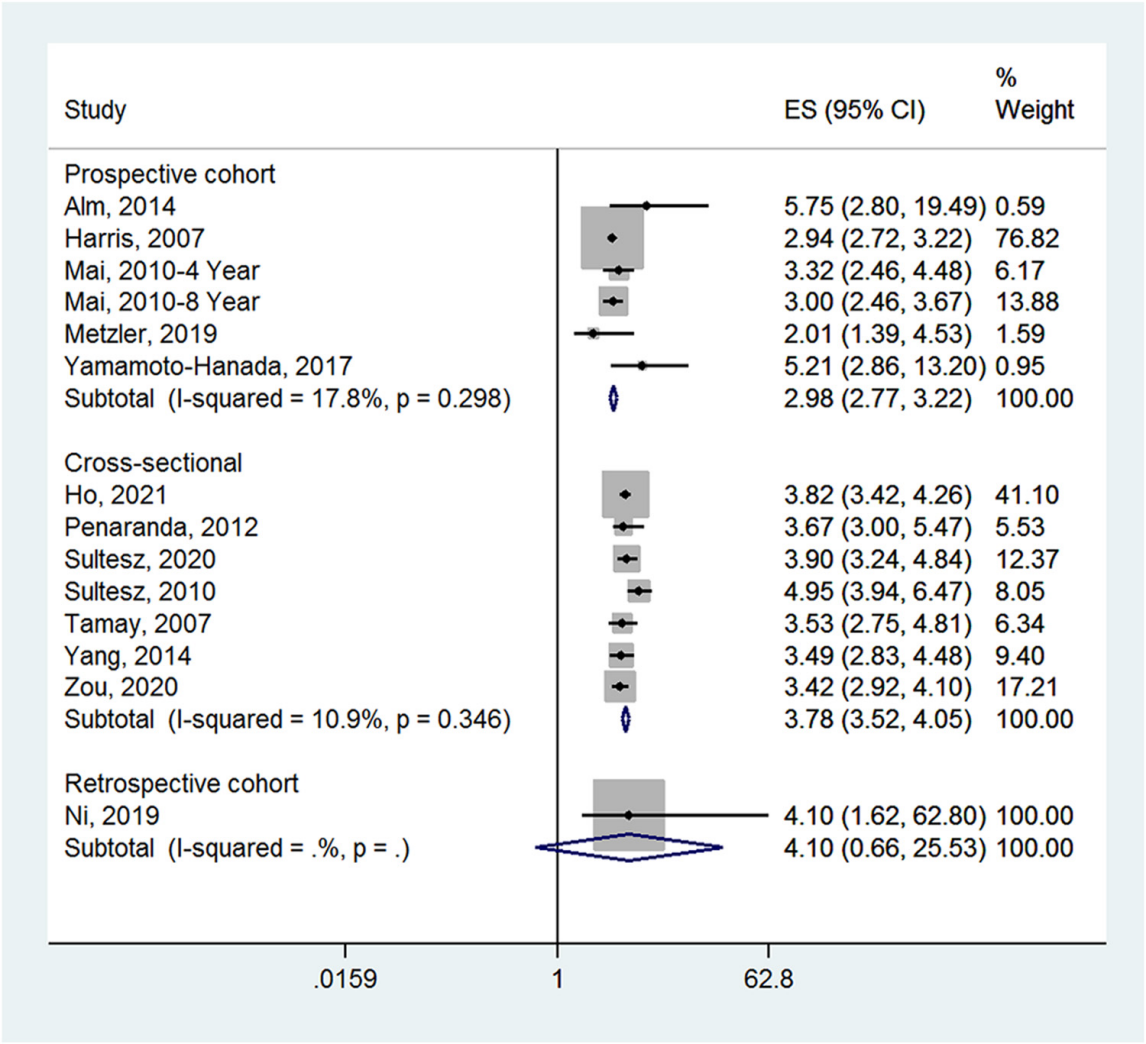
Forest plot for the subgroup analysis by study design in the antibiotic and no medication groups.

**Figure 4:**
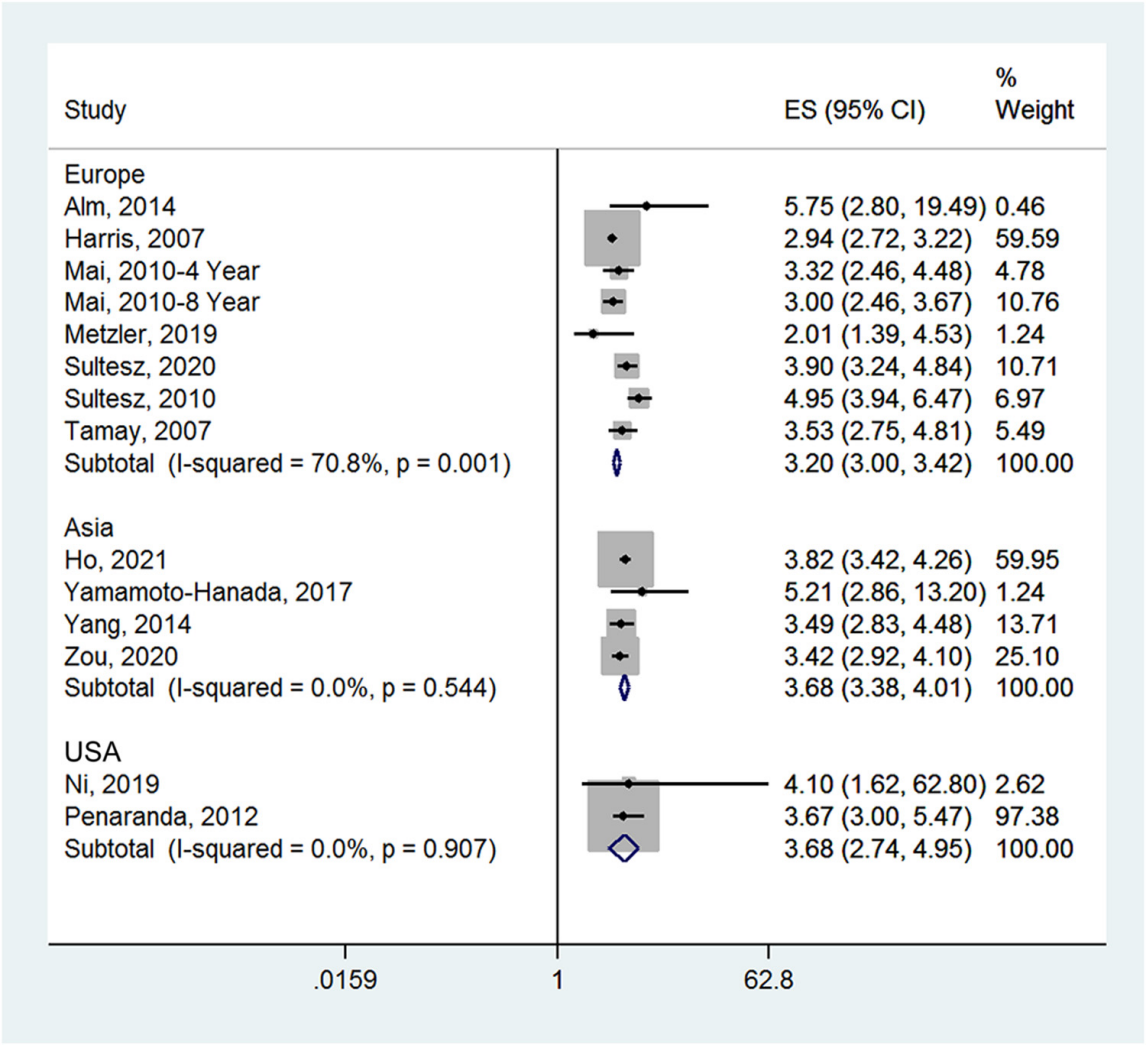
Forest plot for the subgroup analysis by area in the antibiotic and no medication groups.

**Figure 5:**
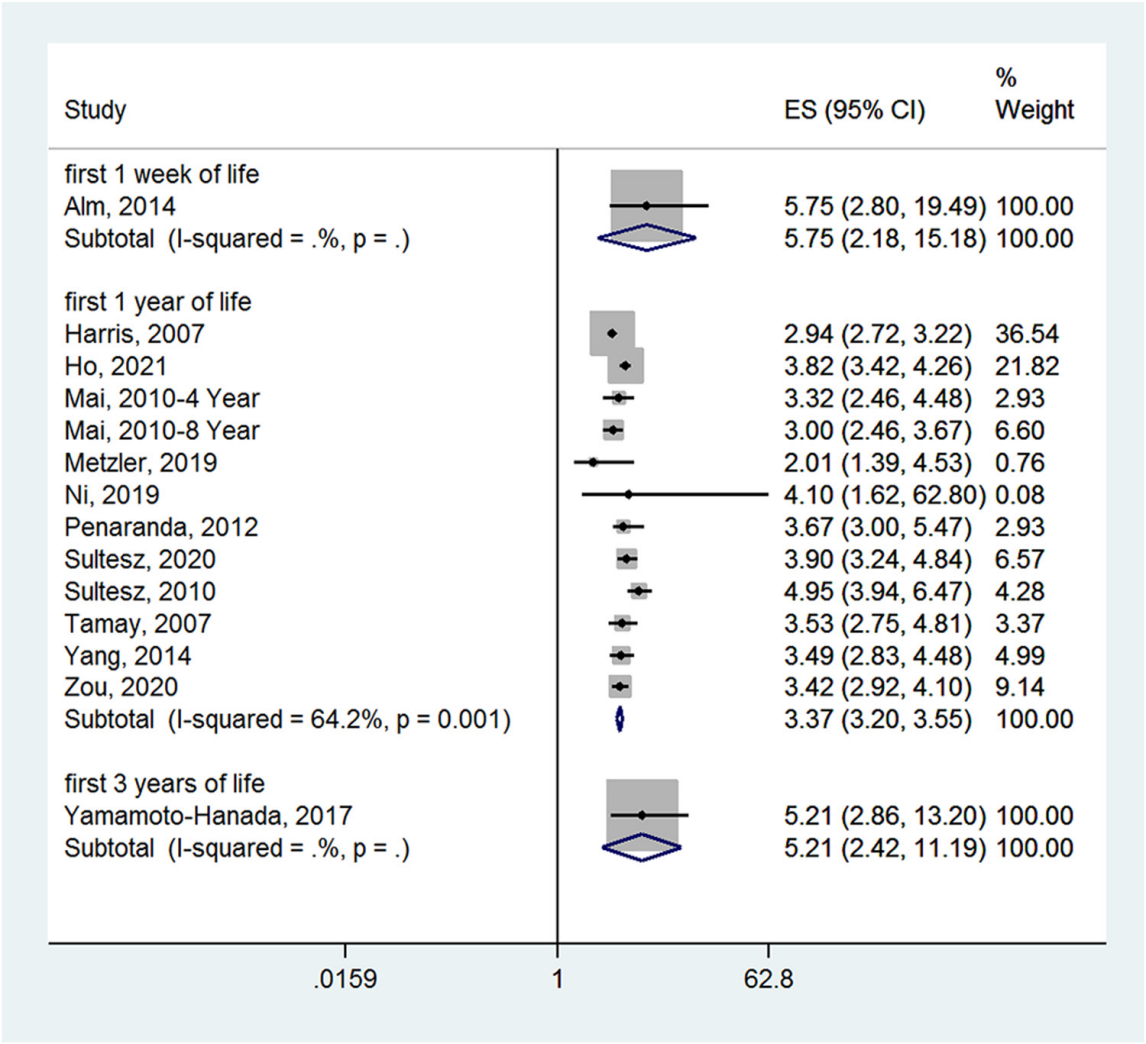
Forest plot for the subgroup analysis by the timing of antibiotic exposure in the antibiotic and no medication groups.

Early-life antibiotics were associated with an increased incidence of allergic rhinitis in children in prospective cohort studies (ES = 2.98, 95% CI: 2.77–3.22; *I* ^2^ = 17.8%, *P*_heterogeneity_ = 0.298) and cross-sectional studies (ES = 3.78, 95% CI: 3.52–4.05; *I* ^2^ = 10.9%, *P*_heterogeneity_ = 0.346), but not in one retrospective cohort study (ES = 4.10, 95% CI: 0.66–25.53) (Figure 3). In Asia, the incidence of allergic rhinitis in the antibiotic group was higher than in the no medication group (ES = 3.68, 95% CI: 3.38–4.01; *I* ^2^ = 0.0%, *P*_heterogeneity_ = 0.544). In Europe and the USA, the incidence of allergic rhinitis in the antibiotic group was also higher than that in the no medication group (ES = 3.20, 95% CI: 3.00–3.42; *I* ^2^ = 70.8%, *P*_heterogeneity_ = 0.001 and ES = 3.68, 95% CI: 2.74–4.95; *I* ^2^ = 0.0%, *P*_heterogeneity_ = 0.907) (Figure 4). Compared with the no medication group, children who received antibiotics in the first 1 week of life (ES = 5.75, 95% CI: 2.18-15.18), first 1 year of life (ES = 3.37, 95% CI: 3.20–3.55; *I* ^2^ = 64.2%, *P*_heterogeneity_ = 0.001), or first 3 years of life (ES = 5.21, 95% CI: 2.42–11.19) had a higher incidence of allergic rhinitis (Figure 5).

### 3.5 Sensitivity analysis

**Figure 6:**
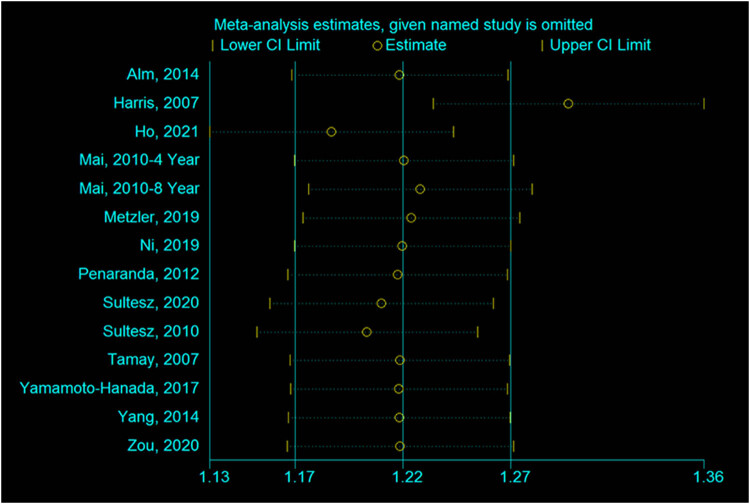
Forest plot for the sensitivity analysis in the incidence of allergic rhinitis in the antibiotic and no medication groups.

As shown in Figure 6, no individual study influenced the results of the meta-analysis.

### 3.6 Publication bias

**Figure 7:**
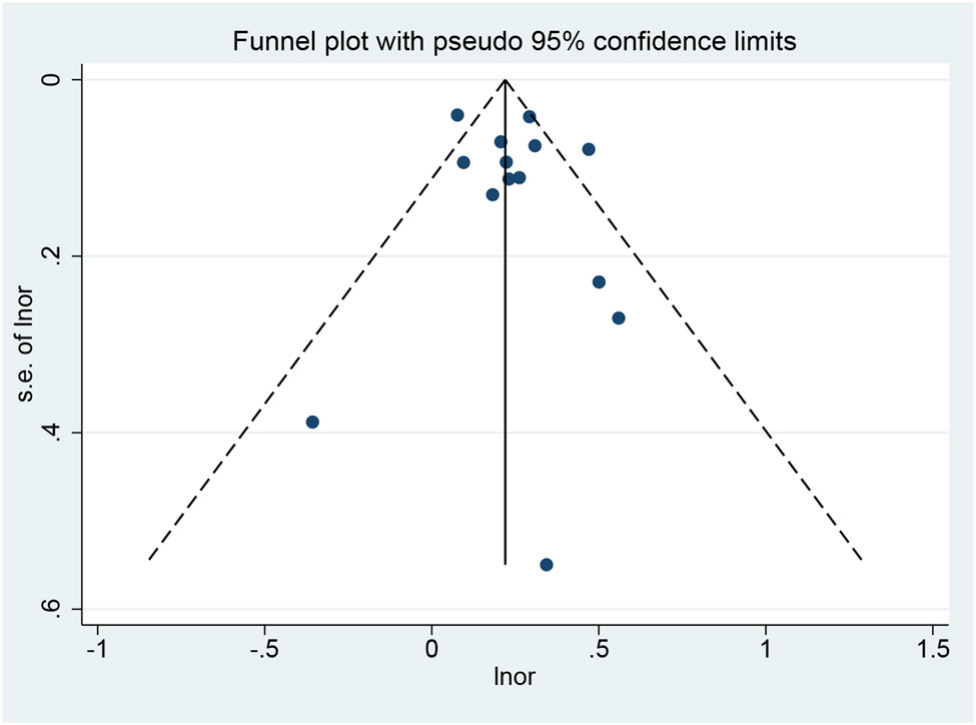
Funnel plot for publication bias.

The funnel plot of the incidence of allergic rhinitis was drawn. All studies are included in the plot. The results showed that the funnel plot had a moderate symmetry and low publication bias (Figure 7).

## 4 Discussion

Previous studies reported conflicting results regarding the early-life use of antibiotics and allergic rhinitis [6,11]. This meta-analysis aimed to investigate the correlation between early-life antibiotic use and allergic rhinitis. The results strongly suggest that the use of antibiotics in early life was associated with allergic rhinitis.

Some antibiotics, especially macrolides, have been found to play an anti-inflammatory role not only by inducing the apoptosis of inflammatory cells but also by regulating the production of pro-inflammatory mediators [18,32,33]. The common side effect of antibiotics is to cause intestinal flora imbalance. The intestinal microbiome has become a research hotspot in the medical field [32]. The formation and changes in the intestinal microbiome exert a direct impact on the growth and development of the fetus from pregnancy to fetal delivery to the early stage of feeding. Allergic rhinitis is one of these allergic diseases. A meta-analysis also revealed an increased incidence of hay fever, eczema, and food allergy in children with early-life antibiotics [34].

In recent years, the incidence of allergic rhinitis has increased significantly in most countries and regions, and the increasing burden of disease has become a global health concern [18,33,35]. More than 500 million people are estimated to suffer from allergic rhinitis worldwide, and the prevalence rate in the USA is 15–30%. In this study, a significant difference was noted in the incidence of allergic rhinitis in the antibiotic and no medication groups. Hence, the incidence of allergic rhinitis in children with early-life antibiotics was higher than in the no medication group. This finding was in agreement with the study observations made by Wilson [36] and Celedon et al. [11] that antibiotics were associated with allergic rhinitis. A Swedish study of 722,767 children also reported similar results [37]. Still, Fsadni et al. [6] reported a limited association between antibiotics and allergic rhinitis in early life. Such discrepancies might be due to the populations being studied, age at exposure, types of antibiotics, dosage, indications, etc. Indeed, Kim et al. [24] showed that the incidence of allergic rhinitis increased with the annual average number of antibiotic prescription days, reaching an OR of 13.4 for >90 days. Tong et al. [29] examined the number of treatments per year and also observed an increased incidence of allergic rhinitis with an increasing number of treatments per year, with an OR of 4.0 for >7 treatments/year. Generally, it appears that penicillin does not increase the incidence of allergic rhinitis, while antibiotics like cephem, macrolides, cephalosporins, and sulfonamides increase the incidence [15,22]. Hence, future studies should be carefully designed to control the effect of such confounders on the incidence of allergic rhinitis in later life.

In the subgroup analyses based on study design, continents, and age at exposure, all subgroups showed a higher incidence in the antibiotic group compared with the no medication group, except for the retrospective cohort study, but there was only one study in that subgroup. These results were consistent with the results by Spector et al. [38].

Nevertheless, the present study has some limitations. First, the quality of a meta-analysis is only as high as the quality of the included studies. Although most studies were of high quality according to the NOS, some lower-quality studies had to be included. In addition, many of the included studies examined antibiotics as the exposure, without considering the different types of antibiotics and their dosages. Therefore, subgroup analyses based on the antibiotics could not be performed here. Second, heterogeneity was high for some analyses, indicating important differences in study populations and treatments. Third, limited publication bias was observed.

In conclusion, this meta-analysis suggests that antibiotics in early life might be associated with allergic rhinitis. Even though antibiotics can be necessary for early life, these results highlight that the use of antibiotics in infants should be carefully weighed to avoid the inappropriate use of antibiotics and cause health issues in later life.

## Appendix

**Table A1:** Search strategies

PubMed		Search strategy	Numbers
**P**	**#1**	"Anti-Bacterial Agents"[Mesh]	413,196
	**#2**	(Agents, Anti-Bacterial) OR (Anti Bacterial Agents) OR (Antibacterial Agents) OR (Agents, Antibacterial) OR (Antibacterial Agent) OR (Agent, Antibacterial) OR (Anti-Bacterial Compounds) OR (Anti Bacterial Compounds) OR (Compounds, Anti-Bacterial) OR (Anti-Bacterial Agent) OR (Agent, Anti-Bacterial) OR (Anti Bacterial Agent) OR (Anti-Bacterial Compound) OR (Anti Bacterial Compound) OR (Compound, Anti-Bacterial) OR (Bacteriocidal Agents) OR (Agents, Bacteriocidal) OR (Bacteriocidal Agent) OR (Agent, Bacteriocidal) OR (Bacteriocide) OR (Bacteriocides) OR (Anti-Mycobacterial Agents) OR (Agents, Anti-Mycobacterial) OR (Anti Mycobacterial Agents) OR (Anti-Mycobacterial Agent) OR (Agent, Anti-Mycobacterial) OR (Anti Mycobacterial Agent) OR (Antimycobacterial Agent) OR (Agent, Antimycobacterial) OR (Antimycobacterial Agents) OR (Agents, Antimycobacterial) OR (Antibiotics) OR (Antibiotic)	1,011,721
	**#3**	#1 OR #2	1,011,721
**Intervention**	**#4**	"Rhinitis, Allergic"[Mesh]	22,630
	**#5**	(Allergic Rhinitides) OR (Rhinitides, Allergic) OR (Allergic Rhinitis)	33,406
	**#6**	#4 OR #5	33,406
**P+I**	**#7**	#3 AND #6	580
**Study type**	**#8**	("Cohort Studies"[Mesh]) OR ((Cohort Study) OR (Studies, Cohort) OR (Study, Cohort) OR (Concurrent Studies) OR (Studies, Concurrent) OR (Concurrent Study) OR (Study, Concurrent) OR (Closed Cohort Studies) OR (Cohort Studies, Closed) OR (Closed Cohort Study) OR (Cohort Study, Closed) OR (Study, Closed Cohort) OR (Studies, Closed Cohort) OR (Birth Cohort Studies) OR (Birth Cohort Study) OR (Cohort Studies, Birth) OR (Cohort Study, Birth) OR (Studies, Birth Cohort) OR (Study, Birth Cohort) OR (Analysis, Cohort) OR (Analyses, Cohort) OR (Cohort Analyses) OR (Cohort Analysis) OR (Historical Cohort Studies) OR (Cohort Studies, Historical) OR (Cohort Study, Historical) OR (Historical Cohort Study) OR (Study, Historical Cohort) OR (Studies, Historical Cohort) OR (Incidence Studies) OR (Incidence Study) OR (Studies, Incidence) OR (Study, Incidence))	2,935,745
**P+I+S**	**#9**	#7 AND #8	164
	**#10**		

## References

[1] Ortqvist AK, Lundholm C, Kieler H, Ludvigsson JF, Fall T, Ye W, et al. Antibiotics in fetal and early life and subsequent childhood asthma: nationwide population based study with sibling analysis. BMJ. 2014;349:g6979. 10.1136/bmj.g6979 PMC4247260 25432937

[2] Candon S, Perez-Arroyo A, Marquet C, Valette F, Foray AP, Pelletier B, et al. Correction: Antibiotics in early life alter the gut microbiome and increase disease incidence in a spontaneous mouse model of autoimmune insulin-dependent diabetes. PLoS one. 2016;11(1):e0147888. 10.1371/journal.pone.0147888 PMC4723038 26799316

[3] Machowska A, Stalsby Lundborg C. Drivers of irrational use of antibiotics in Europe. Int J Environ Res Public Health. 2018;16(1):27. 10.3390/ijerph16010027 PMC6338985 30583571

[4] van den Anker J, Reed MD, Allegaert K, Kearns GL. Developmental changes in pharmacokinetics and pharmacodynamics. J Clin Pharmacol. 2018;58(Suppl 10):S10–25. 10.1002/jcph.1284 30248190

[5] Patel K, Goldman JL. Safety concerns surrounding quinolone use in children. J Clin Pharmacol. 2016;56(9):1060–75. 10.1002/jcph.715 PMC4994191 26865283

[6] Fsadni C, Fsadni P, Fava S, Montefort S. Association of prevalence of rhinitis, atopic eczema, rhinoconjunctivitis and wheezing with mortality from infectious diseases and with antibiotic susceptibility at a country level. Asia Pac Allergy. 2015;5(3):145–55. 10.5415/apallergy.2015.5.3.145 PMC4521163 26240791

[7] Han YY, Forno E, Badellino HA, Celedon JC. Antibiotic use in early life, rural residence, and allergic diseases in Argentinean children. J Allergy Clin Immunol Pract. 2017;5(4):1112–8.e2. 10.1016/j.jaip.2016.12.025 PMC5503768 28174014

[8] Akhouri S, House SA. Allergic Rhinitis. StatPearls. Treasure Island (FL); 2021.

[9] Bousquet J, Anto JM, Bachert C, Baiardini I, Bosnic-Anticevich S, Walter Canonica G, et al. Allergic rhinitis. Nat Rev Dis Primers. 2020;6(1):95. 10.1038/s41572-020-00227-0 33273461

[10] Barberi S, Ciprandi G, Verduci E, D’Auria E, Poli P, Pietra B, et al. Effect of high-dose sublingual immunotherapy on respiratory infections in children allergic to house dust mite. Asia Pac Allergy. 2015;5(3):163–9. 10.5415/apallergy.2015.5.3.163 PMC4521165 26240793

[11] Celedon JC, Fuhlbrigge A, Rifas-Shiman S, Weiss ST, Finkelstein JA. Antibiotic use in the first year of life and asthma in early childhood. Clin Exp Allergy. 2004;34(7):1011–6. 10.1111/j.1365-2222.2004.01994.x 15248843

[12] Yeung SSY, Reijnierse EM, Pham VK, Trappenburg MC, Lim WK, Meskers CGM, et al. Sarcopenia and its association with falls and fractures in older adults: A systematic review and meta-analysis. J Cachexia Sarcopenia Muscle. 2019;10(3):485–500. 10.1002/jcsm.12411 PMC6596401 30993881

[13] Altman DG, Simera I, Hoey J, Moher D, Schulz K. EQUATOR: reporting guidelines for health research. Lancet. 2008;371(9619):1149–50. 10.1016/S0140-6736(08)60505-X 18395566

[14] Higgins JPT, Thomas J, Chandler J, Cumpston M, Li T, Page MJ, et al. Cochrane handbook for systematic reviews of interventions version 6.1. London: Cochrane Collaboration; 2020.

[15] Aversa Z, Atkinson EJ, Schafer MJ, Theiler RN, Rocca WA, Blaser MJ, et al. Association of infant antibiotic exposure with childhood health outcomes. Mayo Clin Proc. 2021;96(1):66–77. 10.1016/j.mayocp.2020.07.019 PMC7796951 33208243

[16] Celedón JC, Litonjua AA, Ryan L, Weiss ST, Gold DR. Lack of association between antibiotic use in the first year of life and asthma, allergic rhinitis, or eczema at age 5 years. Am J Respir Crit Care Med. 2002;166(1):72–5. 10.1164/rccm.2109074 12091174

[17] Harris JM, Mills P, White C, Moffat S, Newman Taylor AJ, Cullinan P. Recorded infections and antibiotics in early life: Associations with allergy in UK children and their parents. Thorax. 2007;62(7):631–7. 10.1136/thx.2006.072124 PMC2117255 17289862

[18] Mai XM, Kull I, Wickman M, Bergström A. Antibiotic use in early life and development of allergic diseases: Respiratory infection as the explanation. Clin Exp Allergy. 2010;40(8):1230–7. 10.1111/j.1365-2222.2010.03532.x 20545711

[19] Metzler S, Frei R, Schmaußer-Hechfellner E, von Mutius E, Pekkanen J, Karvonen AM, et al. Association between antibiotic treatment during pregnancy and infancy and the development of allergic diseases. Pediatric Allergy Immunol. 2019;30(4):423–33. 10.1111/pai.13039 30734960

[20] Mitre E, Susi A, Kropp LE, Schwartz DJ, Gorman GH, Nylund CM. Association between use of acid-suppressive medications and antibiotics during infancy and allergic diseases in early childhood. JAMA Pediatrics. 2018;172(6):e180315. 10.1001/jamapediatrics.2018.0315 PMC6137535 29610864

[21] Ni J, Friedman H, Boyd BC, McGurn A, Babinski P, Markossian T, et al. Early antibiotic exposure and development of asthma and allergic rhinitis in childhood. BMC Pediatrics. 2019;19(1):225. 10.1186/s12887-019-1594-4 PMC6612173 31277618

[22] Yamamoto-Hanada K, Yang L, Narita M, Saito H, Ohya Y. Influence of antibiotic use in early childhood on asthma and allergic diseases at age 5. Ann Allergy Asthma Immunol. 2017;119(1):54–8. 10.1016/j.anai.2017.05.013 28668240

[23] Ho CL, Wu WF. Risk factor analysis of allergic rhinitis in 6–8 year-old children in Taipei. PLoS One. 2021;16(4):e0249572. 10.1371/journal.pone.0249572 PMC8018651 33798255

[24] Kim DH, Han K, Kim SW. Effects of antibiotics on the development of asthma and other allergic diseases in children and adolescents. Allergy Asthma Immunol Res. 2018;10(5):457–65. 10.4168/aair.2018.10.5.457 PMC6082825 30088366

[25] Penaranda A, Aristizabal G, Garcia E, Vasquez C, Rodriguez-Martinez CE, Satizabal CL. Allergic rhinitis and associated factors in schoolchildren from Bogota, Colombia. Rhinology. 2012;50(2):122–8. 10.4193/Rhino11.175 22616072

[26] Sultesz M, Horvath A, Molnar D, Katona G, Mezei G, Hirschberg A, et al. Prevalence of allergic rhinitis, related comorbidities and risk factors in schoolchildren. Allergy Asthma Clin Immunol. 2020;16(1):1. 10.1186/s13223-020-00495-1 PMC7661153 33292450

[27] Sultész M, Katona G, Hirschberg A, Gálffy G. Prevalence and risk factors for allergic rhinitis in primary schoolchildren in Budapest. Int J Pediatric Otorhinolaryngol. 2010;74(5):503–9. 10.1016/j.ijporl.2010.02.008 20211496

[28] Tamay Z, Akcay A, Ones U, Guler N, Kilic G, Zencir M. Prevalence and risk factors for allergic rhinitis in primary school children. Int J Pediatric Otorhinolaryngol. 2007;71(3):463–71. 10.1016/j.ijporl.2006.11.013 17166597

[29] Tong H, Gao L, Deng Y, Kong Y, Xiang R, Tan L, et al. Prevalence of allergic rhinitis and associated risk factors in 6 to 12 years schoolchildren from Wuhan in central China: A cross-sectional study. Am J Rhinol Allergy. 2020;34(5):632–41. 10.1177/1945892420920499 32326719

[30] Yang SI, Lee E, Jung YH, Kim HY, Seo JH, Kwon JW, et al. Effect of antibiotic use and mold exposure in infancy on allergic rhinitis in susceptible adolescents. Ann Allergy Asthma Immunol. 2014;113(2):160–5.e1. 10.1016/j.anai.2014.05.019 24973272

[31] Zou Z, Liu W, Huang C, Sun C, Zhang J. First-year antibiotics exposure in relation to childhood asthma, allergies, and airway illnesses. Int J Environ Res Public Health. 2020;17(16):5700. 10.3390/ijerph17165700 PMC7460111 32784540

[32] Kummeling I, Stelma FF, Dagnelie PC, Snijders BE, Penders J, Huber M, et al. Early life exposure to antibiotics and the subsequent development of eczema, wheeze, and allergic sensitization in the first 2 years of life: The KOALA birth cohort study. Pediatrics. 2007;119(1):e225–31. 10.1542/peds.2006-0896 17200248

[33] Wang JY, Liu LF, Chen CY, Huang YW, Hsiung CA, Tsai HJ. Acetaminophen and/or antibiotic use in early life and the development of childhood allergic diseases. Int J Epidemiol. 2013;42(4):1087–99. 10.1093/ije/dyt121 24062298

[34] Ahmadizar F, Vijverberg SJH, Arets HGM, de Boer A, Lang JE, Garssen J, et al. Early-life antibiotic exposure increases the risk of developing allergic symptoms later in life: A meta-analysis. Allergy. 2018;73(5):971–86. 10.1111/all.13332 29105784

[35] Muc M, Padez C, Pinto AM. Exposure to paracetamol and antibiotics in early life and elevated risk of asthma in childhood. Adv Exp Med Biol. 2013;788:393–400. 10.1007/978-94-007-6627-3_53 23836003

[36] Wilson E. Microbiome: Mouse model shows how antibiotic use in early life leads to weight gain. Chem Eng N. 2013;90(35):9.

[37] Mubanga M, Lundholm C, D’Onofrio BM, Stratmann M, Hedman A, Almqvist C. Association of early life exposure to antibiotics with risk of atopic dermatitis in Sweden. JAMA Netw Open. 2021;4(4):e215245. 10.1001/jamanetworkopen.2021.5245 PMC8085722 33914052

[38] Spector S, Wallace D, Nicklas R, Portnoy J, Blessing-Moore J, Bernstein D, et al. Comments on allergic rhinitis and its impact on asthma (ARIA) guidelines. J Allergy Clin Immunol. 2011;127(6):1641–2. 10.1016/j.jaci.2011.01.071 21496893

